# Liquid Biopsy as a Tool for the Diagnosis, Treatment, and Monitoring of Breast Cancer

**DOI:** 10.3390/ijms23179952

**Published:** 2022-09-01

**Authors:** Ana Julia Aguiar de Freitas, Rhafaela Lima Causin, Muriele Bertagna Varuzza, Stéphanie Calfa, Cassio Murilo Trovo Hidalgo Filho, Tatiana Takahasi Komoto, Cristiano de Pádua Souza, Márcia Maria Chiquitelli Marques

**Affiliations:** 1 Molecular Oncology Research Center, Barretos Cancer Hospital, Teaching and Research Institute, Barretos 14784-400, Brazil; 2 Instituto do Cancer do Estado de São Paulo (ICESP), Universidade de São Paulo, São Paulo 01246-000, Brazil; 3 Barretos Cancer Hospital, Barretos 14784-400, Brazil; 4 Barretos School of Health Sciences, Dr. Paulo Prata–FACISB, Barretos 14785-002, Brazil

**Keywords:** liquid biopsy, precision medicine, breast cancer

## Abstract

Breast cancer (BC) is a highly heterogeneous disease. The treatment of BC is complicated owing to intratumoral complexity. Tissue biopsy and immunohistochemistry are the current gold standard techniques to guide breast cancer therapy; however, these techniques do not assess tumoral molecular heterogeneity. Personalized medicine aims to overcome these biological and clinical complexities. Advances in techniques and computational analyses have enabled increasingly sensitive, specific, and accurate application of liquid biopsy. Such progress has ushered in a new era in precision medicine, where the objective is personalized treatment of breast cancer, early screening, accurate diagnosis and prognosis, relapse detection, longitudinal monitoring, and drug selection. Liquid biopsy can be defined as the sampling of components of tumor cells that are released from a tumor and/or metastatic deposits into the blood, urine, feces, saliva, and other biological substances. Such components include circulating tumor cells (CTCs), circulating tumor DNA (ctDNA) or circulating tumor RNA (ctRNA), platelets, and exosomes. This review aims to highlight the role of liquid biopsy in breast cancer and precision medicine.

## 1. Introduction

Breast cancer (BC) is the most prevalent cancer in women worldwide [1]. Cancer is a complex and heterogeneous disease modulated by genetic, molecular, cellular, tissue-specific, environmental, ethnicity-related, and socioeconomic factors. Because of its global prevalence, many researchers have focused on gaining a better understanding of cancer biology and developing innovative tools for the treatment and diagnosis of BC. Traditional therapies include surgery, chemotherapy, radiotherapy, and immunotherapy [2]. However, there are persistent challenges associated with current breast cancer therapies, such as recurrence [3] and drug resistance [4], which may facilitate tumor metastasis [5,6] and promote cancer progression [7]. An emerging cancer treatment regimen, namely, personalized medicine, is optimized based on a comprehensive understanding of a patient’s individuality with respect to health status and disease stages. Precision medicine includes the analysis of clinicopathological factors and “omics” analysis (genomics, transcriptomics, metabolomics, and proteomics) [8,9].

**Figure 1:**
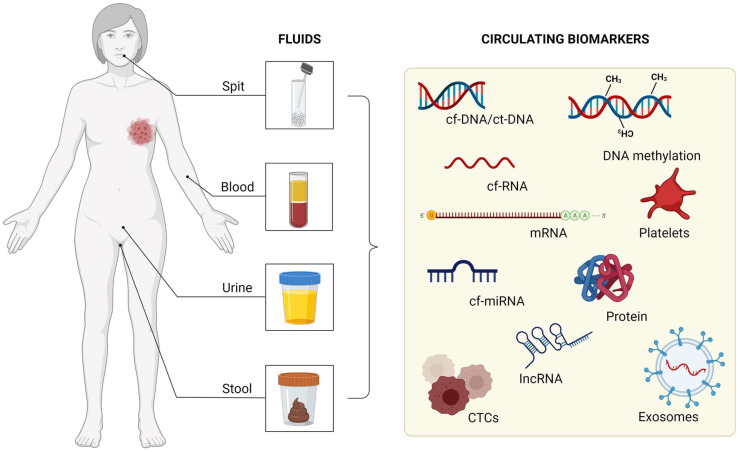
Fluids that can be used as circulating biomarkers.

Precision medicine aims to improve cancer diagnosis and treatment through molecular information that aids in the identification of predictive markers that guide treatment decisions, molecular subtype classification, monitoring of treatment response, and identification of resistance and disease recurrence [10]. Liquid biopsy (LB) has the potential to address the need for personalized therapy through a non-invasive approach [11], and involves analysis of circulating tumor cells (CTCs), circulating tumor DNA (ctDNA), circulating tumor RNA (ctRNA), long non-coding RNAs (lncRNAs), messenger RNA (mRNA), microRNA (miRNA), platelets, tumor-derived extracellular vesicles (microvesicles, exosomes), and proteins, which are released into the urine, serum, saliva, and other biological samples from the primary tumor and/or metastatic deposits (Figure 1) [12]. This is possible as tumor biomarkers are specific and allow accurate distinction of healthy individuals and cancer patients [13,14]. In addition, LB presents some advantages over tumor biopsy, for example, it is easier to access, less painful, and allows the evaluation of tumor heterogeneity as markers from all tumor sites are released into the blood [13,15].

Circulating tumor cells (CTCs) are tumor cells that depart from solid tumor lesions and remain in the bloodstream, and they contain a population of metastatic precursors that are vital for the identification of disease progression [16]. Circulating tumor DNA (ctDNA), another widely studied marker, is a subpopulation of circulating cell-free DNA (cfDNA) in individuals with cancer [17]. While cfDNA refers to DNA released from cells in both healthy and cancerous tissues, ctDNA is a small proportion of the total cfDNA.

Circulating cell-free RNA (cfRNA) molecules have been described, years after cfDNA, in plasma from melanoma patients [18]. With the development of new technologies and more sensitive methods, it has been possible to identify mRNA and miRNA molecules in body fluids, which can be found in ribonucleoprotein complexes, platelets or CTCs, and extracellular vesicles, such as exosomes [19]. cfRNAs that have been touted as one of the hallmarks of cancer can provide information on the tumor gene expression profile, with miRNAs reflecting epigenetic alterations, that have been touted as one of the hallmarks of cancer [20]. One of the main advantages of using miRNAs from LB samples is that they are more stable than mRNAs. Further, miRNAs are tissue specific and regulate several important targets for tumor development and progression [21]. Platelets derived from megakaryocytes can house cytoplasmic RNA secreted by the tumor or captured in circulation through interaction with other cells; such RNA can be translated later into mRNAs, as well as lead to miRNA expression [22]. Similar to platelets, exosomes also contain ctDNA, tumor mRNA, and miRNAs, which are important in the context of LB [23].

Liquid biopsy (LB) can have different applications in clinical practice, including early diagnosis, detection of recurrence, prediction of treatment response (i.e., distinguishing responders and non-responders), longitudinal monitoring during treatment, and drug selection. In addition, it can be used to identify markers that enable patient stratification, leading to personalized therapy. In this review, we highlight the main uses of LB in BC patients. We further focus on circulating biomarkers in biological fluids that can be valuable for cancer research and clinical practice.

## 2. Breast Cancer Screening Using Liquid Biopsy

In the context of a BC diagnosis, mammography is the established gold standard for screening in clinical practice [24]. However, in recent decades, several studies have aimed to develop non-invasive methods for the early detection of BC [25]. Biomarkers based on cfDNA, ctDNA, CTCs, miRNA, lncRNAs, platelets, mRNA, protein, and volatile organic compounds (VOCs) have been previously described and can be derived from the blood (plasma/serum), urine, and saliva. Here, we summarize evidence for the use of non-invasive biomarkers using LB for the early detection of BC (Table 1).

Some studies have identified cfDNA as an early detection biomarker in BC based on analyses of DNA damage and DNA methylation changes. Kamel et al. [26] obtained a DNA integrity index using plasma where patients with confirmed malignancy had significantly greater DNA damage than those with benign breast lesions and healthy controls, and there was a correlation with TNM staging. In another study, Li et al. [27] were the first to assess EGFR and PPM1E promoter methylation status, known to play an important role in cancer progression and tumorigenesis, in plasma using next-generation bisulfite sequencing. In line with what is known about promoter hypermethylation and cancer, they observed that patients with BC had significantly higher methylation levels than healthy controls.

Circulating tumor DNA can be used as a potential biomarker in LB samples to identify specific mutations in BC. Cohen et al. used CancerSEEK, a pan-cancer blood test designed to identify eight types of cancer including BC, to assess mutations in 16 ctDNA genes (including *TP53*, *NRAS*, *CTNNB1*, *PIK3CA*, *KPAS*, *APC*, and *PTEN*). The authors obtained a sensitivity of 33% and specificity of 99% for the plasma detection of BC [28]. Beaver et al. [29] evaluated *PIK3CA* mutations in the plasma of BC patients. *PIK3CA* is an oncogene that mutates at high frequency and is present in approximately 30% of all BCs. The authors demonstrated a sensitivity of 93.3% and a specificity of 100% for detecting early-stage BC.

The detection of CTCs as non-invasive biomarkers for the early diagnosis of BC has yielded promising results. Kruspe et al. [30] developed a rapid and highly sensitive diagnostic method for the detection of CTCs based on nuclease-activated probe technology, which allowed for the discrimination between BC patients and healthy controls by plasma analysis.

MicroRNA molecular profile detection is an opportunity to identify minimally invasive biomarkers for early BC diagnosis. Shimomura et al. [31] evaluated miRNA expression profiles in the serum of BC patients and healthy women. A combination of five miRNAs (miR-1246, miR-1307-3p, miR-4634, miR-6861-5p, and miR-6875-5p) helped to detect BC (sensitivity of 97.3%, specificity of 82.9%, accuracy of 89.7%) and early-stage BC individuals (98.0% sensitivity for carcinoma in situ). Erbes et al. [32] carried out the first study to identify differential circulating miRNA profiles (miR-21, miR-125b, miR-451, and miR-155) in the blood and urine of BC patients, allowing them to specifically discriminate between patients with local BC and healthy women. In addition, this study reported the reliability, reproducibility, and robustness of analyses involving urine samples. Furthermore, Hirschfeld et al. [33] identified the differential expression of four ct-miRNAs (miR-424, miR-423, miR-660, and let7-i) in the urine of BC patients, successfully distinguishing BC patients from healthy controls.

A case-control study identified a molecular signature of miRNAs as LB biomarkers for each molecular subtype. This suggests that the LB approach using molecular biomarkers can be used for routine BC screening [34].

Plasma exosome-derived lncRNAs are abundant in many types of cancer, including BC, and thus, they are potential tumor biomarkers [35,36,37]. Zhong et al. [37] analyzed serum exosomal lncRNA H19, an oncogene associated with cell proliferation, invasion, and apoptosis, and a biomarker previously reported for monitoring BC progression [38]. The authors observed that exosomal H19 expression was significantly upregulated in the serum of patients with BC as compared twith that in patients without malignancy, indicating that this biomarker was a promising diagnostic indicator and was superior to standard markers.

Tumor-educated platelets (TEPs) can be used as biomarkers for BC diagnosis using blood samples. Best et al. [39] conducted a pan-cancer study involving six tumor types, including BC. The primary tumor location was correctly identified with 71% accuracy, and since each molecular subtype induced different stimuli that affected platelet profile, the analyses, based on TEP profiles, successfully distinguished between BC patients who had HER2-amplified, PIK3CA-mutant, or triple-negative phenotypes.

Messenger RNAs (mRNAs) and proteins are promising early BC biomarkers that can be identified in biological samples, including saliva. Zhang et al. [40] established that nine biomarkers, eight mRNAs (*S100A8*, *GRIK1*, *GRM1*, *H6PD*, *IGF2BP1*, *CSTA*, *MDM4*, and *TPT1*), and a CA6 protein (carbonic anhydrase VI) were able to distinguish between BC patients and healthy controls. A diagnostic accuracy of 92% (sensitivity of 83% and specificity of 97%) was observed. Another study showed that the levels of CA125 (cancer antigen 125) and sFas proteins were significantly increased in the saliva of BC patients, and that they were able to successfully discriminate between groups (BC patients vs. healthy controls). The combination of these biomarkers demonstrated a sensitivity of 67.5% and specificity of 66.7%. CA125 and sFas are relevant tumor biomarkers, since CA125 is a glycoprotein with antiadhesive properties, and sFas is a cell surface receptor, which inhibits apoptosis and contributes to tumor progression [41].

Volatile organic compounds (VOCs) have been identified as potential biomarkers in BC screening and are detectable in biological samples, including urine. Kure et al. found a combination of 2-butanone and 2-propanol, which are compounds produced during mechanisms associated with tumorigenesis. Volatile organic compounds (VOCs) were highly effective in detecting early-stage BC and they achieved a sensitivity of 93.3% and specificity of 83.3% [42].

## 3. Use of Liquid Biopsy to Aid in Drug Selection

Drug resistance has become the biggest obstacle to the success of cancer therapies, accounting for more than 90% of deaths in cancer patients receiving traditional chemotherapy or new targeted drugs [43]. Resistance mechanisms include increased metabolism of xenobiotics, increased drug efflux, growth factors, increased DNA repair capacity, and genetic factors (genetic mutations, amplifications, and epigenetic alterations) [44]. LB can be used to determine the most effective and accurate treatments and may be a promising non-invasive method; therefore, we summarize the main studies related to treatment decisions based on LB analysis (Table 2).

With the developments in research, the prediction of treatment response to drug treatment based on LB has become possible. For instance, Di Cosimo et al. [45] found that increases in miR-148a-3p and miR-374a-5p in the blood were associated with a pathological complete response (pCR) after trastuzumab-based neoadjuvant therapy, indicating that these miRNAs could be used as predictive biomarkers. Moreover, using Gene Ontology (GO) and KEGG analyses, they found that these miRNAs were associated with cell metabolism regulation and AMPK and MAPK signaling.

In another study, it was verified that miR-503 increased in the plasma of patients with BC after neoadjuvant treatment, which occurred as a consequence of the upregulation of exosomes released from endothelial cells after treatment with paclitaxel and epirubicin. Interestingly, while upregulation of miR-503 was observed in patients who received neoadjuvant chemotherapy, no changes were observed in patients treated with surgery alone [46]. This miRNA may contribute to the direct effects of taxane and anthracycline therapy and could be used as a predictive biomarker.

Circulating tumor DNA (ctDNA) is another important tool that can be used to monitor the development and treatment of cancer. In a randomized phase III study (PALOMA-3), in which the CDK4/6 inhibitors palbociclib and fulvestrant were tested in a group of women (521 patients) with advanced BC (estrogen receptor-positive BC and HER2-negative BC), there were changes in PIK3CA ctDNA levels in plasma samples after 15 days of treatment with palbociclib and fulvestrant. This result indicates that early detection of ctDNA can provide potent biomarkers for CDK4/6 inhibitors drugs [47].

One study analyzed samples from 38 patients with early-stage TNBC, who received neoadjuvant treatment with a combination of cisplatin and rucaparib. The presence of ctDNA in all patients who had recurrence demonstrated that next-generation ctDNA sequencing may be a useful strategy for predicting recurrence after neoadjuvant treatment in patients with TNBC [48].

Circulating tumor cells (CTCs) also stand out as important markers that can help to identify chemoresistance, which is related to a lower prognosis in early BC. [49]. In a study with a total of 444 BC patients (stages I–III) who received adjuvant chemotherapy, and had a follow-up of 10 months with adjuvant chemotherapy regimens, the patients presenting CK-19 (cytokeratin-19) mRNA-positive CTCs experienced reduced disease-free survival (DFS) and overall survival (OS) after treatment [50]. In addition, they analyzed the presence of CTCs in patients with CB (stage I–III), independent of HER-2 status, and observed that post trastuzumab administration, 75% of patients had no detectable CTCs for CK19 mRNA as compared with the observation arm (17.9%). Another study analyzed the blood samples of 437 patients with early breast cancer, before and after adjuvant chemotherapy, and observed a greater reduction in CTCs positive for CK19 mRNA [51]. In contrast, a study that aimed to assess whether trastuzumab decreased the detection rate of CTCs in women with high-risk, HER-2 non-amplified, early BC analyzed 1318 HER-2-negative patients after adjuvant treatment and screened for CTCs in the blood. From those, 7.2% presented as CTC positive, and were divided into two groups: observation or trastuzumab administration. However, they did not observe a decrease in CTCs after trastuzumab treatment [52].

Many studies have been conducted to evaluate new therapeutic methods using LB to better detect miRNAs, ctDNA, and CTCs, as a tool for real-time monitoring of disease progression and treatment efficacy to improve personalized medicine and treatment decision making.

## 4. Monitoring Residual Disease Using Liquid Biopsy Biomarkers during Treatment

Disease monitoring using LB has been increasingly investigated for various malignancies, including BC [53,54]. LB can be used to stratify patients with variable risk of recurrence during therapy [55], based on CTC analysis or factors derived from circulating tumors, in particular, ctDNA or exosomes [56].

The evolution of highly sensitive LB-based assays has allowed us to detect and characterize minimal residual disease (MRD), in order to identify the presence of tumor cells that have disseminated from a primary tumor to distant organs in patients who do not show clinical or radiological signs of metastasis, or residual tumor cells abandoned after local therapy, eventually leading to local recurrence [56]. In this context, LB assays can be used to monitor MRD, helping in the discovery of new drugs that can effectively eliminate or control residual tumor cells in patients with high-risk disease recurrence after primary therapy. The results of studies, published in the last 7 years, on patients with early or advanced BC have demonstrated that many biomarkers can be used to monitor the response to treatment, including analyses involving ctDNA, CTC counts, circulating endothelial cells (CEC), exosomal microRNA (exo-miRNA) expression, circulating IL-8, fecal metabolites, and even analysis of platelet aggregation (Table 3). Similar correlations have been reported for other tumor types, including colorectal cancer [57,58] and bladder cancer [59].

### 4.1. Circulating ctDNA

Circulating tumor DNA (ctDNA) detection in biological fluids has been widely discussed over the years, and recent improvements in ctDNA sequencing and analysis technology have allowed for its use in MRD detection in many types of tumors, such as lung, breast, colon, pancreatic, and bladder cancers. Overall, MRD could assist in the management of patients with cancer at all stages, including monitoring response and resistance to treatment. The concentration of detectable ctDNA is determined by the tumor type, tumor burden, and other biological processes, such as therapy resistance. Among the technologies currently used for ctDNA detection, in this review, we identified whole-genome sequencing (WGS), which can identify somatic mutations, as well as copy number variations (CNVs), and structural rearrangements [70], a technique based on droplet digital polymerase chain reaction (ddPCR) and targeted digital sequencing (TARDIS).

Understanding the technology used and its ability to detect the desired target is essential for assessing the clinical significance of ctDNA [70]. For this reason, many researchers have focused on techniques that are fast, sensitive, and cost-effective, such as ddPCR, which is among the most widely used techniques to date [64]. This method is based on the distribution of ctDNA samples in from hundreds to millions of droplets of water-in-oil emulsions [71]. The advantages of ddPCR include its excellent sensitivity for identifying mutations and low cost for absolute quantification. However, this method can detect only known variants and analyze only a limited number of variants. One of the studies that detected ctDNA by using TARDIS could identify residual disease in patients with early and locally advanced-stage BC with excellent accuracy after neoadjuvant treatment. This detection method identified ctDNA in all patients, with 0.11% median variant allele frequency (VAF) before therapy [62].

### 4.2. Platelets, CTC, and CEC

Studies have linked CTC and CEC counts to RD in patients undergoing treatment for BC. Darga et al. analyzed blood from metastatic BC patients and healthy donors for CTC and platelet PD-L1 with a phycoerythrin-labeled anti-human PD-L1 monoclonal antibody, using the CellSearch^®^ assay [65]. They identified PD-L1 expression in metastatic BC patients on both CTC and platelets in an independent fashion. These data suggest that CTC and platelet PD-L1 expression could play a role in predicting which patients should receive immune checkpoint inhibition, and also, as a pharmacodynamic biomarker during treatment.

Pierga et al. evaluated CTC and CEC in 137 patients with locally advanced BC using the CellSearch ^®^ system [66]. The study found that at baseline, 55 patients had detectable CTC (39%). After four cycles of chemotherapy, a dramatic drop in CTC to a rate of 9% (*p* < 0.01) was observed, with a pCR rate of 40%. The mean follow-up duration was 43 months. CTC detection (≥1 CTC/7.5 mL) at baseline was associated with lower 3-year DFS (39% vs. 70% for patients without CTC, *p* < 0.01, HR 2, 80) and shorter 3-year OS (*p* < 0.01). However, the CEC level at baseline or variations during treatment responded to treatment monitoring.

### 4.3. Exo-miRNAs, IL-8, and Fecal Metabolomics

Exosomal microRNAs (exo-miRNAs) have recently been investigated in cancer studies. Aberrant miRNA expression has already been identified and characterized in a range of biological samples, such as tissues, serum, plasma, CTCs, and exosomes, and their role in the development of new biomarkers for BC has been explored [72]. Although, few studies have focused on the detection of exo-miRNAs for monitoring the response to therapy in BC [73], Todorova et al. recently investigated the ability of circulating exo-miRNAs to predict pCR in BC patients treated with neoadjuvant chemotherapy (NAC), using next-generation sequencing (NGS) technology [67]. The authors found that three miRNAs predicted pCR in all analyzed samples (miR-30b, miR-328, and miR-423) before NAC. In addition, they identified that exo-miRNAs could contribute to monitoring response to neoadjuvant treatment. After the first dose of NAC, pCR was predicted by exo-miR-141, whereas exo-miR-34a, exo-miR182, and exo-miR-183 predicted DR. However, these miRNAs still need to be validated in a larger cohort. Therefore, further studies are needed to assess the robustness and reproducibility of exo-miRNAs and to independently validate exo-miRNA signatures.

Proinflammatory cytokines are also targets for biomarker research for BC, as their effects on the tumor microenvironment may result in tumor proliferation, survival, and chemoresistance in malignant diseases [74,75]. Tiainen et al. performed an exploratory analysis of multiple plasma cytokines and circulating proteins and found that the most evident predictor was interleukin-8 (IL-8), because the majority of patients (n = 35, 60%) with lower levels of IL-8 throughout treatment had better OS. Thus, low levels of IL-8 during chemotherapy may help to identify patients with prolonged survival [68].

Metabolomics is a new, state-of-the-art method with demonstrated effectiveness in numerous studies, providing information on biological systems complementary to that provided by other “omics” approaches [76]. Metabolomics provides a powerful tool for the discovery of clinically relevant biomarkers [69]. This approach also allows for the identification of metabolites that relate to the modulation of responses to anticancer treatments, which is called pharmacometabolomics [77]. Zidi et al. performed a pioneering study to identify and characterize specific profiles of fecal metabolites in patients with BC after chemotherapy, and established a non-invasive metabolomics approach to improve the monitoring of patients with BC [69]. They demonstrated that chemotherapy modulated the fecal metabolomic profile of patients with BC. Therefore, these data provide interesting insights that can complement and improve clinical tools for monitoring BC, using a multitude of samples, including stool samples.

## 5. Prediction of Treatment Response and Early Detection of Relapse

Several types of cells or molecules, such as CECs, CTCs, peripheral blood mononuclear cells (PMBCs), circulating cancer stem-like cells (sCSCs), cfDNA, ctDNA, mRNA, miRNA, and exosomes in blood samples, as well as metabolic markers in urine samples, can predict treatment response and/or early detection of disease relapse in BC patients (Table 4).

### 5.1. Circulating Cells as Biomarkers

Several cell types with the potential to provide information about a patient’s treatment response, as well as their chance of recurrence, have already been identified. Among these cells, CECs, CTCs, sCSCs, and PMBCs stand out. A recent study evaluated treatment response by scoring CECs, in blood samples from patients with locally advanced BC, who underwent NAC (epirubicin, cyclophosphamide, and docetaxel). The number of CECs increased after the first cycle and decreased after eight cycles of NAC as compared with the baseline pretreatment samples. The study also evaluated chromosomal alterations in these CECs and found aneuploid CECs in all patients, and these numbers increased post NAC. Therefore, a better understanding of aneuploid CECs and their relationship with cancer may help elucidate the development of chemotherapy resistance and metastasis processes [78].

Circulating tumor cells (CTCs) are the main circulating cells currently being studied in the context of LB and BC. This review identified 16 studies that assessed the presence of CTCs and their use as predictive biomarkers for treatment response or relapse. These studies associated a high CTC count with shorter progression-free survival (PFS), DFS and/or OS [79,80,81,82,83,84], resistance to different therapies [80,84,85], worse outcome [82,84,85], disease relapse [82,85], and metastasis [86]. Some studies have also evaluated CTC counts with different phenotypes, such as mesenchymal CTCs (mCTCs) and epithelial CTCs (eCTCs). In these studies, a high count of mCTCs as compared with eCTCs, was associated with progressive disease [80,86] and metastatic development [86,87], indicating their clinical importance in therapeutic resistance. Another study showed high expression of chemoresistance-associated genes (MRP1, MRP2, MRP4, MRP5, MRP7, MDR1, and ERCC1) in CTCs [85].

In addition, the expression of markers in CTCs has been evaluated and compared with that in PMBCs to provide answers about metastatic disease. CTCs positive for CD47 and PD-L1 markers were detected in patients with de novo metastatic disease, but not in those with early disease. In addition, CD47^+^ CTCs and PD-L1^+^ CTCs correlated with disease progression and reduced PFS and OS [88]. Furthermore, TLR4^+^ and pSTAT3^+^ levels were assessed. High rates of pSTAT3^+^ CTCs were detected in early-stage patients, and high rates of TLR4^+^ CTCs were detected in metastatic patients, indicating shorter PFS. In addition, both molecules are present during disease progression and are associated with shorter OS. Among PMBCs, TLR4^+^ was associated with visceral metastases, and TLR4^+^/pSTAT3^−^ PBMCs had a high risk of death in metastatic patients [89]. This metabolic classification of CTCs may help identify aggressive CTC subpopulations and provide new targeted therapies.

In the context of cells as biomarkers, cCSCs have been shown to correlate with CTCs. A low cCSC count is related to superior tumor response, PFS, and OS, all of which are potential prognostic factors [90]. In addition to cells, the expression of mRNA and miRNAs in CTCs has been studied. TP53 expression was assessed in CTCs positive for EpCAM, KRT19, and MUC1 markers during cancer treatment, and its expression was associated with stage IV disease at the initial diagnosis. KRT19^+^ CTCs were associated with shorter PFS, OS, and early progression. These results show an evolutionary change in CTC gene expression that could be involved in treatment predictive genes during tumor progression [91]. Another study evaluated a panel of five miRNAs (miR-21-5p, miR-222-3p, miR-221-3p, miR-155-5p, and miR-105-5p) based on their relationship with cell proliferation and cancer progression. The evaluation was performed at the time of diagnosis and after four cycles of doxorubicin/cyclophosphamide in patients with local and metastatic disease; miR-21 was associated with larger tumors at diagnosis, miR-222 was associated with the proliferation marker Ki-67, and miR-221 was found at lower levels after treatment in patients with lymph node metastasis. Moreover, these miRNAs were associated with CTC counts, where miR-21, miR-222, and miR-155 levels were positively correlated with CTCs. In addition, higher levels of miR-21 and miR-155 after treatment were associated with a high number of CTCs before treatment [72].

The association between circulating DNA molecules and CTCs has been assessed in two studies. The first study correlated CTC count with cfDNA and conventional BC markers (CA15-3 and alkaline phosphatase [AP]). The results showed a strong correlation between high CTC counts, cfDNA, CA15-3, and AP, with worse outcomes in metastatic patients. Additionally, high CTC and AP levels are predictors of progressive disease. Circulating cell-free DNA (cfDNA) is a treatment response predictor that identifies responding and non-responding patient groups [92]. The second study evaluated CTCs and ctDNA mutations (*ESR1* and *PIK3CA*) and correlated their expression with TK1 (thymidine kinase-1) activity at the beginning of endocrine therapy, after four weeks of treatment, and at the time of progressive disease. High CTC counts and TK1 levels were found at baseline and were related to lower PFS, and may be useful as a prognostic marker and for monitoring early response to endocrine therapy [93].

### 5.2. Nucleic Acids as Biomarkers

Along with cells, isolated nucleic acids have also been studied for their potential role as cancer biomarkers. Several studies have evaluated circulating DNA as a predictor of treatment response, disease progression [94], shorter PFS [95,96], and worse patient outcomes [97,98]. In addition, four studies evaluated mutations and/or gene expression in ctDNA. Trastuzumab-resistant processes were assessed in patients who progressed, and mutations in *TP53*, *SETD2*, *CDK12*, *EGFR*, and *NF1* were detected in most patients. Other somatic mutations were found in patients with stable disease (*RNF43*, *NTRK1*, *NF1*, *ERBB2*, and *PAK3*) and were present at high frequencies in patients with disease progression. In addition, the *ERBB2* expression level was lower in patients who benefited from trastuzumab than in those who developed resistance [94].

Another study evaluated the number of detected alterations and mutant allele frequencies in ctDNAs. The mutant allele frequency decreased, and the number of detected alterations increased in progressive cases. In addition, *TP53*, *PIK3CA*, ERBB2, *MET*, *EGFR*, and *ESR1* were the most represented genes [97]. KRAS mutations in ctDNA were associated with treatment resistance to palbociclib and fulvestrant. Most patients were negative for mutKRAS ctDNA at baseline, and some patients turned positive after the start of treatment. After a follow-up in eighteen months, all mutKRAS ctDNA patients showed disease progression. In addition, the median PFS was better in patients with wild-type KRAS ctDNA [95]. Mutations in *ERBB2*, *ESR1*, *PIK3CA*, *MYC*, and cyclin D1 variants were also detected in patients who underwent palbociclib treatment during disease progression [96].

It is possible to assess treatment response or predict who will not benefit from specific cancer treatments using miRNA expression [67,99,100,101]. The genes miR21-5p, miR-100-5p, miR-125b-5p, miR-126-3p, miR-375, and miR-424-5p are miRNAs related to BC and the pathways targeted by the drug, dovitinib. The expression of these genes was evaluated in response to dovitinib and aromatase inhibitors. In patients with tumor resistance to dovitinib, miR-125b, miR-126, miR-375, miR-424, and miR-100 were downregulated post treatment as compared with patients with stable disease or sensitive tumors [99].

Some studies have evaluated responses to NAC. One study evaluated non-responsive patients with lower expression levels of miR-185, miR-4283, miR-5008, and miR-3613 and higher expression levels of miR-1302, miR-4715, and miR-3144 [100]. In addition, one study evaluated miRNA expression related to radioresistance, stemness, DNA repair, and metastasis, where miR-21, miR-10b, miR-221, miR-210, and miR-142 expression levels increased after radiotherapy (RT). When comparing expression during and after RT, they found decreased expression levels of miR-21, mir-15b, and miR-182 and an increased expression level of miR-221 [101].

Moreover, RNA molecules have been studied as extracellular vesicle long RNA (exLR) and exosomes, with the potential to predict treatment response [102] and disease status [103]. ExLR was investigated in patients who received NAC (paclitaxel and/or doxorubicin), and it was found that 2573 exLRs were differentially expressed in patients who achieved pCR as compared with those with residual disease. A gene set enrichment analysis (GSEA) revealed the upregulation of exLR from the steroid biosynthesis pathway in patients with residual disease, and the exMSMO1 (methylsterol monooxygenase 1) level could distinguish patient outcomes [102]. Heat shock protein-70 (HSP70)-exosomes were investigated in blood and urine, and their levels were found to be increased in the blood of patients with metastasis. However, no differences were found between the metastatic and non-metastatic urine samples [103].

### 5.3. Metabolic Biomarkers

Urine samples have been investigated for their potential in acting as predictive biomarkers of treatment response. In this context, the metabolites released in the urine have been assessed. N-telopeptide of type I collagen (NTX), a bone metabolism marker, was evaluated in patients before, during, and after zoledronic acid treatment. The results showed that NTX levels varied according to extraskeletal involvement, and persistently high levels during treatment were associated with a doubled risk of death [104].

11-Dehydro-thromboxane (TX) B2, a marker of in vivo platelet activation, was also assessed in urine samples. Pre-surgical urinary samples showed a gradual increase in patients with carcinoma in situ, invasive carcinoma, local recurrence, and distant metastases. In addition, high levels of 11-dehydro-TXB2 have been associated with a worse pathological response to NAC. Therefore, increased oxidative stress can induce lipid peroxidation, which may contribute to platelet activation and worse outcomes [105].

## 6. Applications of Liquid Biopsy in Clinical Practice

Advances in the development of increasingly precise and specific LB platforms over the past decade have led to regulatory approvals for blood-based tests that enable precision treatment for patients with advanced diseases, including BC (Table 5).

### 6.1. Circulating Tumor Cells

The first Food and Drug Administration (FDA)-approved LB assay, which covers BC patients [106], is the CellSearch System^®^ platform (Veridex, Raritan, NJ, USA), which is designed for magnetic enrichment, fluorescent labeling, and CTC detection [107].

**Table 5:** FDA-approved tests using liquid biopsy for breast cancer.

Test	Biomarkers	Method	Ref.
CellSearch	CTCs	CellSearch System	[108]
Guardant360	ctDNA	NGS	[109]
FoundationOne Liquid	ctDNA	NGS	[110]

CTCs, circulating tumor cells; ctDNA, circulating tumor DNA; NGS, next-generation sequencing.

In a cohort of 177 patients with metastatic BC, the results showed that ≥5 CTCs in 7.5 mL were associated with a significantly shorter OS and PFS [111]. Subsequently, several studies using the CellSearch platform showed that positive CTC counts were associated with poor prognosis in metastatic BC [112,113,114]. More recently, the same technique was tested in another study to monitor the response to palbociclib in advanced hormone receptor-positive BC, which showed that among 46 patients, those with detectable CTCs after the first cycle of palbociclib had lower PFS, and patients with ≥5 CTCs at disease progression had a shorter time to treatment failure [84]. Since the CellSearch System^®^ only detects CTCs that express cytokeratin and EpCAM, other techniques have been developed to overcome its limitations. The RareCyte platform, which consists of the AccuCyte^®^ sample preparation system and CyteFinder instrument, detects CTCs, despite the EpCAM status [115] and shows similar analytical and prognostic value [116]. Two other marker-independent technologies have been tested (CellSieve™ filters and ScreenCell^®)^ to identify CTC clusters with promising results [117]. Although different technologies have been developed, the CellSearch System^®^ is the most established, and is still the only FDA-approved CTC detection method for BC.

### 6.2. Circulating Tumor DNA

Recently, ctDNA analysis using NGS has been introduced in clinical practice. These tests can detect genomic changes in solid tumors. The FDA has approved two tests for ctDNA detection: Guardant360 CDx (Guardant Health, Inc., Redwood City, CA, USA), which can detect changes in more than 60 different cancer-related genes [109], and FoundationOne^®^ Liquid CDx, which can identify mutations or changes in more than 300 genes, supporting treatment in an individualized way, considering the unique characteristics of each type of cancer [110].

In the metastatic scenario, ctDNA has demonstrated a better correlation with tumor burden and prediction of treatment response as compared with carbohydrate antigen 15.3 (CA15.3) and CTCs [118]. A recent meta-analysis including 1127 metastatic BC patients showed poor PFS and OS associated with the presence of ctDNA [119]. Another ctDNA application is the identification of genetic mutations with clinical relevance for cancer treatment. The plasMATCH trial evaluated the accuracy of ctDNA testing based on the agreement between ctDNA and tissue mutation status and concluded that ctDNA testing has an important role in identifying rare targetable mutations, such as *PIK3CA*, *ESR1*, *HER2*, *AKT1,* and *PTEN* [120]. Recent studies have used ctDNAs to evaluate the mechanisms of resistance to CDK4/6 inhibitors. Mutations were associated with poor prognosis, such as mutations in CDK4/6-Rb pathway genes, including *CDK4* and *CDK6*, *CCND1*, *CDKN2A*, and *RB1* [121], *ESR1* mutations [122], *TP53* alterations, and *FGFR* gains [123]. Studies have shown divergent results, and the investigated biomarkers have not yet been used in clinical practice [124].

In the neoadjuvant setting, ctDNA clearance analysis has shown promising results in predicting pCR [62]; its persistence was associated with recurrence and poor response to NAC [63]. This suggests that monitoring of this biomarker could provide early information on treatment efficacy. However, the role of ctDNA analysis in NAC has not been well established, and there is controversial information about it in the literature. A recent meta-analysis did not find a significant association between ctDNA detection and pCR [125]. Therefore, further elucidation is needed before this biomarker can be introduced in clinical practice in patients receiving NAC.

### 6.3. Other Serum Markers

CA15.3 and carcinoembryonic antigen (CEA) are serum markers often used in clinical practice to monitor response to cancer therapy in metastatic BC patients. The sensitivity values of CA15.3 and CEA are approximately 70% and 50%, respectively, for predicting disease progression, when above the 95th percentile of healthy individuals [126]. These markers also correlate with tumor burden and are particularly useful when correlating to clinical evaluation in patients with non-measurable or non-assessable lesions by RECISTv1.1 [127]. There are several limitations in the use of CA15.3 and CEA in metastatic BC follow-up; these serum markers have low specificity and conflicting results in studies. Finally, the National Comprehensive Cancer Network (NCCN) recommends that isolated rising tumor markers should not be used to define disease progression and should be considered in patients with bone-dominant metastasis in association with patient symptoms (category 2A) [128].

## 7. Final Considerations

With advances in molecular technologies in recent years, the detection and analysis of body fluids have been the subject of many studies, demonstrating that LB provides the opportunity to assist decision making and, therefore, aid personalized treatment of breast tumors. Not considered as a substitute, but as a combined analysis, Lennon et al. described the use of LB followed by positron emission tomography/computed tomography (PET-CT) in patients who tested positive for ctDNA, as an accurate alternative to diagnose different types of malignancies, the site, and extent of disease. However, further studies are needed to assess the clinical utility, risk, and cost-effectiveness of the tests [129]. LB has different advantages over tissue biopsy; tissue biopsy provides information about the tumor at a certain time and place, while LB has the potential to assess the spatial and temporal heterogeneity of the tumor in a non-invasive manner, enabling the monitoring of subclonal evolution through serial collections [130].

Disease monitoring is important, since each clinical subtype has different preferential sites of metastatic involvement, and there are no diagnostic tools capable of correctly predicting the site of recurrence. A retrospective study analyzed a cohort of patients with metastatic breast cancer to understand the biological characteristics of the disease in real time according to different sites of metastasis, aiming at a more personalized therapeutic approach. The results suggested that changes detected in LB could be used to develop predictive models to monitor organs at a higher risk of metastasis [131].

The use of CTCs has already been approved by the FDA for BC, therefore, for this reason, its clinical utility on defining treatment strategy has been explored. A randomized clinical trial demonstrated the role of CTCs on treatment choice and concluded that CTCs could be a reliable biomarker method to guide the choice between chemotherapy and endocrine therapy as a first-line treatment in metastatic breast cancer [132]. In contrast, another randomized clinical trial failed to demonstrate the clinical utility of CTC monitoring [133].

Current research suggests the potential use of CTCs [134] and ctDNA [135] as an alternative to assess hormonal and HER2 status in advanced breast cancer, as LB is a non-invasive and easy-to-repeat sampling approach. However, this strategy lacks clinical validation and is currently not recommended by major guidelines.

**Figure 2:**
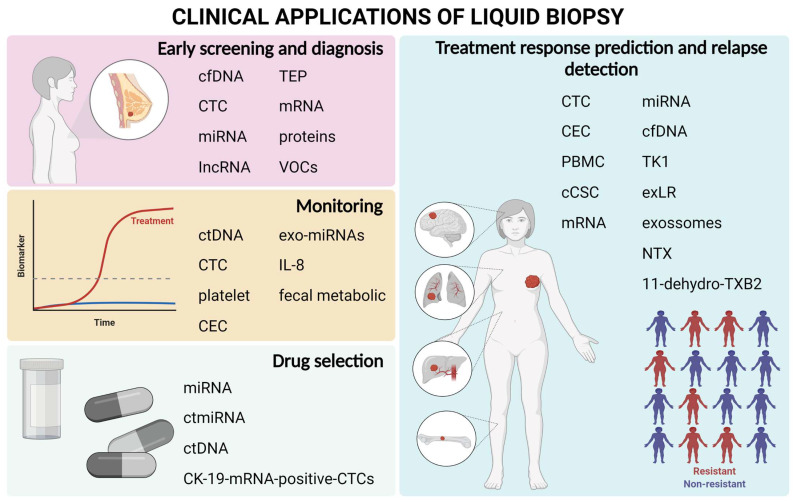
Main clinical uses of liquid biopsy with the biomarkers highlighted in the review.

The main perspectives for the clinical use of LB include early detection, detection of metastases, real-time monitoring, and treatment selection for resistance to treatment, among others (Figure 2).

Although there are many limitations and challenges, clinical trials have extensively explored the use of LB in the setting of neoadjuvant treatment of patients with BC, since, with the use of biomarkers that use “omics” technologies, they can help in the development of new medications and in identifying and monitoring patients who will respond to and benefit from treatment [9].

The high cost of the technique, attributable to sensitivity and precision requirements, limits the application [136]. Another challenge is the analytical and computational aspects, as they require multiple analyses with large cohorts to obtain results that can be significant, and thus, reproducible [10]. As described, the application of LB in the oncological context is already a reality since the FDA has approved assays to detect genetic alterations in cfDNA and CTCs for breast tumors.

In conclusion, despite the efforts of the scientific community, most LB assays still lack evidence and clinical validity, and their use is limited for research purposes. Further controlled and randomized clinical trials that compare the use of LB with the gold standard, are required to validate and evaluate the benefits of its use in clinical practice.

## Figures and Tables

**Table 1:** Summary of potential non-invasive biomarkers using liquid biopsy for early detection of breast cancer.

Study, Year	Sample	N	Stage of Disease	Biomarker	Sensitivity (%)	Specificity (%)	Accuracy (%)	Detection Method	Ref.
Kamel et al., 2016	Plasma	95	I–IV	cf-DNA	85.3	100	-	RT-qPCR	[26]
Li et al., 2016	Plasma	86	I–II	cf-DNA	75.6–94.2	30.4–53.3	66–75	Microfluidic PCR and Bisulfite Sequencing Technology	[27]
Cohen et al., 2018	Plasma	54	I–III	ct-DNA	33	99	73	Multiplex-PCR, NGS and CancerSEEK	[28]
Beaver et al., 2014	Plasma	29	I–III	ct-DNA	93.3	100	96.7	ddPCR	[29]
Kruspe et al., 2017	Plasma	29	IV	CTCs	-	-	-	RT-qPCR	[30]
Shimomura et al., 2016	Serum	1206	I–IV	miRNA	97.3	82.9	89.7	Microarray and RT-qPCR	[31]
Erbes et al., 2015	Serum and urine	24	Early	miRNA	83.3	87.5	88.7	RT-qPCR	[32]
Hirschfeld et al., 2020	Urine	69	Early	miRNA	98.6	100	99.9	RT-qPCR	[33]
Zhong et al., 2020	Serum	50	I–IV	lncRNA	87	70.6	87	RT-qPCR	[37]
Best et al., 2015	Blood	39	I–IV	TEPs	-	-	71	mRNA sequencing	[39]
Zhang et al., 2010	Saliva	40	I–IV	mRNA and proteins	83	97	92	Microarray, RT-qPCR, and immunoblot	[40]
López-Jornet et al., 2021	Saliva	91	I–IV	Proteins	67.5	66.7	-	Biochemical analyses	[41]
Kure et al., 2021	Urine	110	I–II	VOCs	93.3	83.3	88.3	GCMS	[42]

cfDNA, cell-free DNA; CTCs, circulating tumor cells; miRNA, microRNA; lncRNA, long non-coding RNAs; TEPs, tumor-educated platelets; VOCs, volatile organic compounds; mRNA, messenger RNA; NGS, next-generation sequencing; RT-qPCR, reverse transcription quantitative real-time PCR; GCMS, gas chromatography–mass spectrometry; ddPCR, droplet digital polymerase chain reaction.

**Table 2:** Summary of studies demonstrating the use of liquid biopsy to aid in drug selection.

Study, Year	Sample	Subtype	Role	Drug	Biomarker	Ref.
Cosimo el al., 2020	Blood	HER2+	Predictive biomarker	Trastuzumab	miRNA and ct-miRNA	[45]
Boyy et al., 2015	Plasma	NA	Therapeutic target/prognostic indicator	Paclitaxel and epirubicin	miRNA	[46]
O’Leary B et al., 2018	Plasma	ER+, HER2-	Predictive biomarker	Palbociclib and fulvestrant	ctDNA	[47]
Chen Y et al., 2017	Plasma	TN	Predictive biomarker	Cisplatin and rucaparib	ctDNA	[48]
Ignatiadis et al., 2007	Blood	ER+, ER-, TN, HER2+, and ER+/HER2-	Predictive biomarker	Fluorouracil, epirubicin, cyclophosphamide, docetaxel, methotrexate	CK-19 mRNA-positive CTCs	[50]
Xenidis et al., 2009	Blood	ER+, ER-, TN, HER2+, and ER+/HER2-	Predictive biomarker	Fluorouracil, epirubicin, cyclophosphamide, docetaxel, methotrexate	CK-19 mRNA-positive CTCs	[51]

miRNAs, microRNAs; ct-miRNA, circulating tumor miRNA; ctDNA, circulating tumor DNA; CK-19 mRNA-positive CTCs: cytokeratin-19 (CK-19) mRNA-positive circulating tumor cells; ER+, estrogen receptor positive; ER-, estrogen receptor negative; TN, triplo-negative; HER2+, human epidermal growth factor receptor-2 positive; HER2-, human epidermal growth factor receptor-2 negative; NA, not available.

**Table 3:** Studies demonstrating the biomarkers for monitoring response during treatment in breast cancer using liquid biopsy.

Study, Year	Sample	N	Stage of Disease	Biomarker	Detection Method	Ref.
Garcia-Murillas et al., 2015	Plasma	55	Early	ctDNA	ddPCR	[60]
Kodahl el al., 2018	Serum	66	Advanced disease	ctDNA	ddPCR	[61]
McDonald et al., 2019	Plasma	33	Early and locally advanced disease	ctDNA	TARDIS	[62]
Magbanua et al., 2021	Plasma	291	Early	ctDNA	WGS	[63]
Olsson et al., 2015	Plasma	20	Early	ctDNA	WGS e ddPCR	[64]
Darga et al., 2021	Blood and platelet	124	Advanced disease	CTC sand platelet PD-L1	CellSearch System^®^	[65]
Pierga et al., 2017	Blood	137	Locally advanced disease	CTCs and CECs	CellSearch System^®^	[66]
Todorova et al., 2022	Plasma	20	Early and advanced disease	exo-miRNAs	NGS	[67]
Tiainen et al., 2019	Plasma	58	Advanced disease	IL-8	ELISA	[68]
Zidi et al., 2021	Stool	8	Early	Fecal Metabolic	NMR Spectroscopy	[69]

ctDNA, circulating tumor DNA; CTCs, circulating tumor cells; CECs, circulating endothelial cells; exo-miRNAs, exosomal microRNAs; WGS, whole-genome sequencing; ddPCR, droplet digital polymerase chain reaction; TARDIS, targeted digital sequencing; NGS, next-generation sequencing; NMR, nuclear magnetic resonance.

**Table 4:** Summary of potential non-invasive biomarkers using liquid biopsy for prediction of treatment response and early detection of relapse.

Study, Year	Sample	N	Stage of Disease	Biomarker	Detection method	Ref.
Rodriguéz-Martínez et al., 2019	Blood	53	Not available	CTCs/miRNA	Immunocytochemistry/RT-qPCR	[72]
Ma et al., 2020	Blood	41	Locally advanced disease	CECs	SE-iFISH	[78]
Pierga et al., 2012	Blood	267	Metastatic disease	CTCs	CellSearch System^®^	[79]
Yu et al., 2013	Blood	41	Metastatic disease	CTCs	Microfluidic HB chip/NGS	[80]
Horimoto et al., 2018	Blood	22	IV	CTCs	Microfluidic chip	[81]
Costa et al., 2020	Blood	54	Metastatic disease	CTCs	CellSearch System^®^	[82]
Brisotto et al., 2020	Blood	31	Metastatic disease	CTCs	MBA/CellSearch System^®^	[83]
Galardi et al., 2021	Blood	46	Not available	CTCs	CellSearch System^®^/ddPCR	[84]
Jakabova et al., 2021	Blood	20	Early and locally advanced disease	CTCs	MetaCell/q-PCR	[85]
Chen et al., 2020	Blood	64	I–IV	CTCs	RNA-ISH	[86]
Zhou et al., 2020	Blood	89	I–IV	CTCs	Flow cytometry/immunofluorescence/RT-qPCR	[87]
Papadaki et al., 2020	Blood	198	Early and metastatic disease	CTCs/PBMC	Ficoll–Hypaque density-gradient centrifugation/Immunofluorescence	[88]
Papadaki et al., 2022	Blood	199	Early and metastatic disease	CTCs/PBMC	Ficoll–Hypaque density-gradient centrifugation/immunofluorescence	[89]
Lee et al., 2019	Blood	48	IV	CTCs/cCSCs	Flow cytometry	[90]
Aaltonen et al., 2017	Plasma	36	Metastatic disease	CTCs/mRNA	CellSearch System/Multiplex q-PCR	[91]
Fernandez-Garcia et al., 2019	Plasma	194	Metastatic disease	CTCs/cfDNA	CellSearch System/RT-qPCR	[92]
Bonechi et al., 2018	Plasma	32	Metastatic disease	CTCs/ctDNA/TK1	CellSearch System/ddPCR	[93]
Chen et al., 2020	Plasma	31	I–IV	ctDNA	NGS	[94]
Raimondi et al., 2021	Plasma	106	Metastatic disease	ctDNA	ddPCR	[95]
Chin et al., 2022	Plasma	33	Metastatic disease	ctDNA	NGS/ddPCR	[96]
Gerratana et al., 2021	Plasma	107/48	IV	ctDNA	NGS/ddPCR	[97]
Wang et al., 2021	Plasma	273	Not available	ctDNA	NGS	[98]
Shivapurkar et al., 2017	Plasma	12	Metastatic disease	miRNA	RT-qPCR	[99]
Salvador-Coloma et al., 2020	Plasma	34	Early or locally advanced disease	miRNA	Microarray	[100]
Griñán-Lisón et al., 2021	Blood	60	Not available	miRNA	q-PCR	[101]
Su et al., 2021	Plasma	172	I–IV	exLR	NGS	[102]
Chanteloup et al., 2020	Plasma/urine	20	Not available	Exosomes/CTCs	BLI/ELISA/NTA/CellSearch System	[103]
Ferreira et al., 2016	Urine	71	Metastatic disease	NTX	ELISA	[104]
Ferroni et al., 2017	Urine	115	I–III	11-dehydro-TXB2	Radioimmunoassay	[105]

CECs, circulating endothelial cells; SE-iFISH, subtraction enrichment and immunostaining-fluorescence in situ hybridization; CTCs, circulating tumor cells; NGS, next-generation sequencing; MBA, metabolic-based assay; ddPCR, droplet digital polymerase chain reaction; qPCR, quantitative polymerase chain reaction; RNA-ISH, RNA in situ hybridization; RT-qPCR, reverse transcriptase quantitative real-time polymerase chain reaction; PBMC, peripheral blood mononuclear cell; cCSCs, circulating cancer stem-like cells; mRNA, messenger RNA; miRNA, microRNA; cfDNA, cell-free DNA; ctDNA, circulating tumor DNA; TK1, thymidine kinase-1; exLR, extracellular vesicle long RNA; BLI, biolayer interferometry; ELISA, enzyme-linked immunosorbent assay; NTA, nanoparticle tracking analysis; NTX, N-telopeptide of type I collagen; 11-dehydro-TXB2, 11-dehydrothromboxane B2.

## Data Availability

No new data were created or analyzed in this study. Data sharing is not applicable for this study.
